# Supplementary material for: HIV-1 Envelope Glycoprotein Amino Acids Signatures Associated with Clade B Transmitted/Founder and Recent Viruses
Source: Viruses. 2019 Nov 1;11(11):1012. doi: 10.3390/v11111012 (PMC6893788; doi:10.3390/v11111012)
Supplement: Supplementary file 1 [file viruses-11-01012-s001.zip › Table S1. Defining of the different timeline categories of HIV-1 infection status and referred nomenclatures.docx]

**Table S1:** Samples description: defining timeline categories of HIV-1 infection and referred nomenclatures.

| **Infection category** | **Virus type** | **Fiebig stages** | **Infection duration** | **Biomarkers** | **Sample derived from** |
| --- | --- | --- | --- | --- | --- |
| Acute infection | Transmitted/founder viruses (TF) | Fieb. 1-2 | 14-21 days | HIV-RNA+ and p24 Ag+, Western blot -, HIV antibody- | Acutely infected individuals |
| Early infection | Recent viruses (RC) | Fieb. 3-5 | ≤ 136 days according RITA testing, *Sherir B. et al. 2016.* | HIV p24 Ag-, Western blot +, HIV antibody +, qualified as recent by RITA testing | Recently infected individuals |
| Chronic infection | Chronic viruses (CH) | Established | > 6 months according RITA testing, *Sherir B. al. 2016.* | HIV antibody + | Chronically infected individuals |

***Table S1*** *presents the defining timeline categories of HIV-1 status and referred nomenclatures frequently used in manuscript. Abbreviations: RITA: Recent infection testing algorithm; Ag: Antigen; (-): negative, (+); positive; WB: Western Blot; Fieb: Fiebig stage*
