# Supplementary material for: HIV-1 Envelope Glycoprotein Amino Acids Signatures Associated with Clade B Transmitted/Founder and Recent Viruses
Source: Viruses. 2019 Nov 1;11(11):1012. doi: 10.3390/v11111012 (PMC6893788; doi:10.3390/v11111012)
Supplement: Supplementary file 1 [file viruses-11-01012-s001.zip › Table S2. HIV-1 envelope subregions and domains locations according HXB2 numbering.docx]

**Table S2: Summary of the HIV-1 envelope subregions and domains locations according HXB2 envelope (gp160) numbering.**

| **Order** | **Envelope subdomain** | **HXB2 Position** | **Alignment position** | **Sequence length** |
| --- | --- | --- | --- | --- |
|  | **GP120** | **1-511** | **1-627** | **512** |
| 1 | SP | M1-A30 | 1-42 | 42 |
| 2 | C1 | T31–K130 | 43-145 | 103 |
| 3 | V1 | C131-C157 | 146-204 | 59 |
| 4 | V2 | S126-C196 | 141-270 | 130 |
| 5 | C2 | N197-N295 | 271-370 | 100 |
| 6 | Loop D | V275-T283 | 350-358 | 9 |
| 7 | V3 | C296-C331 | 371-408 | 38 |
| 8 | C3 | N332–Y384 | 409-466 | 58 |
| 9 | V4 | C385-C418 | 467-520 | 54 |
| 10 | CD4 binding loop | S364-H374 | 445-456 | 12 |
| 11 | C4 | R419–G459 | 521-561 | 41 |
| 12 | V5 | N460-R469 | 566-577 | 12 |
| 13 | C5 | P470–R511 | 578-627 | 50 |
|  | **GP41** | **512-856** | **628-977** | **345** |
| **1** | **ECD** | **A512-N677** | **628-797** | **170** |
|  | FP | A512-G527 | 628-644 | 17 |
|  | FPPR | T529-L545 | 646-662 | 17 |
|  | NHR | S546-L581 | 663-698 | 36 |
|  | PFD | A582-Q590 | 699-707 | 9 |
|  | Loop | Q591-I622 | 708-742 | 35 |
|  | CHR | W623-E659 | 743-779 | 37 |
|  | MPER | W673-F683 | 795-805 | 11 |
| 2 | **TMD or MSD** | **N677- S 716** | **N794 -S833** | 40 |
| 3 | **CD** | **N706-L856** | **N823-977** | 151 |
|  | EC (GYSPL) | G711-L715 | 832-835 | 5 |
|  | HIR/KE (P—S) | P724-S745 | 844-866 | 25 |
|  | NA (C---L) | C764-R770 | 885-891 | 7 |
|  | LLP-2 | Y768-R788 | 889-909 | 21 |
|  | LLP-3 | G789-L815 | 910-936 | 27 |
|  | LLP-1 (R---E | R828-855L | 949-976 | 28 |
|  | EC (LL) | L855-L856 | 976-977 | 2 |

**Abbreviations.** GP: Glycoproteins; ECD: Ectodomain; TMD: Transmembrane domain, CD: Cytoplasmic domain; MSD: membrane-spanning domain; EC: endocytosis; HIR: highly immunogenic region; KE: Kennedy epitope, NA: NF-κB activation; LLP: lentivirus lytic peptide, FPPR: Fusion peptide proximal region. Refs [15, 37, 41, 42, 44, 61-63, 81].
