# Supplementary material for: HIV-1 Envelope Glycoprotein Amino Acids Signatures Associated with Clade B Transmitted/Founder and Recent Viruses
Source: Viruses. 2019 Nov 1;11(11):1012. doi: 10.3390/v11111012 (PMC6893788; doi:10.3390/v11111012)
Supplement: Supplementary file 1 [file viruses-11-01012-s001.zip › Table S5. Descriptive statistics of HIV-1 envelope variable regions characteristics.docx]

**Table S5.** Descriptive statistics of HIV-1 envelope variable regions characteristics.

| **Parameters** | **Infection status** | |  | **Logistic Regression analysis** | | | | | | | | |
| --- | --- | --- | --- | --- | --- | --- | --- | --- | --- | --- | --- | --- |
|  |  |  |  | CH vs RC | | CH vs TF | | RC vs TF | | CH vs RC vs TF | | |
| HIV-1 Env variables regions characteristics | CH (N=105) | RC (N=28) | TF (N=98) | OR | P | OR | P | OR | P | OR | P |  |
| V1 Glyco Num, median (Range) | 4 (4,5) | 4 (4,6) | 4 (4,5) | 0.6543105 | 0.165 | 0.9486806 | 0.796 | 1.507161 | 0.195 |  |  |  |
| V2 Glyco Num, median (Range) | 2 (2,2) | 2 (2,2.5) | 2 (2,2) | 0.8293438 | 0.655 | 0.5753516 | 0.064 | 0.7065884 | 0.437 |  |  |  |
| V1+V2 Glyco Num, median (Range) | 7 (6,7) | 7 (6,7.5) | 7 (6,7) | 0.7234489 | 0.248 | 0.758415 | 0.151 | 1.080748 | 0.793 |  |  |  |
| V3 Glyco Num, median (Range) | 2 (2,2) | 2 (2,3) | 2 (2,2) | **2.032284** | **0.057** | **0.5856403** | **0.026** | **0.3794798** | **0.004** |  |  |  |
| V4 Glyco Num, median (Range) | 5 (4,5) | 4 (4,5) | 5 (4,5) | 1.128342 | 0.681 | 1.148025 | 0.457 | 1.026063 | 0.926 |  |  |  |
| V5 Glyco Num, median (Range) | 2 (1,2) | 2 (1.5, 2) | 2 (1, 2) | 1.09692 | 0.824 | 0.9732193 | 0.923 | 0.8946218 | 0.783 |  |  |  |
| V1 Length, median (Range) | 30 (28, 34) | 29.5 (26.5, 38.5) | 31 (27, 34) | 1.044247 | 0.212 | 0.9907996 | 0.729 | 0.9538506 | 0.167 |  |  |  |
| V2 Length, median (range) | 43(41, 46) | 42.5 (41, 46) | 42 (40, 45) | 0.9889286 | 0.846 | 0.9732534 | 0.453 | 0.9834442 | 0.756 |  |  |  |
| V1+V2 length, median (Range) | 72 (68, 77) | 73 (66.5, 80) | 72 (67, 76) | 1.032432 | 0.309 | 0.9837571 | 0.458 | 0.9638805 | 0.188 |  |  |  |
| V3 Length, median (Range) | 37 (37, 37) | 37 (37, 37) | 37 (37, 37) | 1.129842 | 0.695 | 1.215851 | 0.543 | 1.186667 | 0.808 |  |  |  |
| V4 Length, median (Range) | 31 (29, 33) | 32 (30,34) | 31.5 (30, 33) | 1.099581 | 0.158 | 1.050612 | 0.317 | 0.9167963 | 0.280 |  |  |  |
| V5 Length, median (Range) | 15 (12, 14) | 15 (13, 15) | 13 (12, 14) | **1.44851** | **0.004** | 0.9790804 | 0.813 | **0.6537583** | **0.003** |  |  |  |
| V3 net Charge, median (Range) | 5 (3, 6) | 4 (3, 5.5) | 4 (3, 5) | 0.9419158 | 0.640 | **0.8264031** | **0.038** | 0.8718934 | 0.347 | **-.1655425** | **0.040** |  |

Table S5 presents the summary statistics of HIV-1 envelope variables regions characteristics (numbers of N-glycosylation sites, loop lengths and V3 net charge for transmitted/founder (TF) viruses compared to recent (RC) and chronic (CH) ones. Statistically significant results were shown in red in table.
